# Mid-Life Cardiorespiratory Fitness, Obesity, and Risk of Atrial Fibrillation: The Cooper Center Longitudinal Study

**DOI:** 10.1016/j.jacadv.2022.100040

**Published:** 2022-05-31

**Authors:** Ambarish Pandey, Benjamin Willis, Carolyn E. Barlow, David Leonard, Vijay Agusala, Laura F. DeFina, Jarett D. Berry

**Affiliations:** ^a^Division of Cardiology, Department of Internal Medicine, UT Southwestern Medical Center, Dallas, Texas, USA; ^b^Research Division, The Cooper Institute, Dallas, Texas, USA

**Keywords:** atrial fibrillation, body mass index, cardiorespiratory fitness, obesity

## Abstract

**Background:**

Lower cardiorespiratory fitness (CRF) and higher body mass index (BMI) are associated with a higher risk of myocardial infarction and heart failure. However, the independent contribution of these lifestyle factors to the risk of atrial fibrillation (AF) is less well established.

**Objectives:**

The purpose of this study was to evaluation the association between midlife CRF, BMI, and risk of AF in older age.

**Methods:**

This study included 18,493 participants without AF who underwent assessment of CRF (estimated using the maximal treadmill time) and BMI in middle age and had Medicare coverage after the age of 65 years. The association among midlife CRF, BMI, and risk of AF was assessed by fitting a proportional hazards intensity model to the failure time data with adjustment for potential confounders. The association between changes in CRF and BMI in middle age and the risk of AF was also assessed in the subset of participants with repeat CRF assessments.

**Results:**

Among 18,493 participants (79% men), a higher midlife BMI was significantly associated with a higher risk of AF independent of CRF levels and other potential confounders (hazard ratio per 1-kg/m^2^: 1.05; 95% confidence interval: 1.03-1.06). Lower midlife CRF was also associated with higher risk of AF (hazard ratio per 1 MET higher CRF: 0.98; 95% confidence interval: 0.96-0.99). However, this association was attenuated and not significant after further adjustment for BMI. Change in CRF on follow-up was also not associated with the risk of AF after adjustment for other confounders.

**Conclusions:**

The association between low fitness and AF was primarily driven by differences in BMI. In contrast, obesity was independently associated with excess AF risk.

Atrial fibrillation (AF) is the most common cardiac arrhythmia encountered in clinical practice affecting up to 5.1 million adults in the United States. AF burden is expected to double over the next 20 years.^1^ Furthermore, AF is predominantly a disease of the elderly, with the prevalence of AF increasing by 5% annually among individuals aged ≥65 years.^1^ AF is associated with a high comorbidity burden, health care costs, and excess mortality risk.^2^^,^^3^ As the incidence of AF continues to rise,^1^ effective preventive strategies to reduce the burden of AF in the aging population are needed.

Lifestyle risk factors such as physical inactivity, low cardiorespiratory fitness (CRF), and obesity have been previously associated with a higher risk of cardiovascular diseases (CVD) such as myocardial infarction and heart failure (HF).^4^, ^5^, ^6^ Obesity has also been associated with a higher risk of AF in prior cohort studies.^7^, ^8^, ^9^ However, the association between physical activity (PA) and incident AF has been inconsistent^10^, ^11^, ^12^, ^13^, ^14^, ^15^, ^16^ possibly due to heterogeneity in self-reporting PA measurements, which may bias study findings toward no association. In contrast, CRF, an objective measure of habitual PA, is a stronger predictor of CVD than self-reported PA.^17^, ^18^, ^19^, ^20^, ^21^ While recent studies have evaluated the relationship between CRF and AF risk, the observed associations have been inconsistent.^22^, ^23^, ^24^, ^25^, ^26^, ^27^, ^28^, ^29^ Moreover, few studies have assessed the independent contribution of BMI and CRF to the risk of AF. Furthermore, the association of changes in exercise capacity, BMI, and AF risk is not well established. Accordingly, we evaluated the association between CRF and BMI measures and changes in midlife and the risk of AF after the age of 65 years among participants in the Cooper Center Longitudinal Study (CCLS).

## Methods

### Study population

The CCLS is an ongoing study that follows up patients of a preventive health clinic in Dallas, Texas (Cooper Clinic). The details of the study design and protocol have been previously well described.^30^^,^^31^ The majority of participants are male, Caucasian, and from the middle and upper socioeconomic levels. Patients are able to self-refer to the clinic or may be referred by their primary care physician or their employer. The initial cohort included 23,194 CCLS participants with a qualifying midlife exam who later entered a surveillance period of Medicare administrative claims data from 1999 to 2009. Qualifying exams occurred before the surveillance period and had complete data on required covariates, including BMI, systolic blood pressure, cholesterol, fasting blood glucose, and a modified-Balke treadmill exercise test. Exclusions consisted of 338 participants with a history of myocardial infarction or stroke, 500 participants who entered Medicare before the age of 65 years due to disability or renal dialysis, 3,106 participants without traditional fee-for-service Medicare, and 757 participants diagnosed with AF before the surveillance period, leaving a final cohort of 18,493 CCLS participants (Supplemental Figure 1).

In a subset of study participants (N = 7,435), a repeat treadmill exercise test was performed an average of 2.3 years after the qualifying examination. No CCLS participant was excluded from the present analysis based on their maximal treadmill test results. Participants in the CCLS provide informed consent for their data to be used for research, and the CCLS is approved annually by the Cooper Institute Institutional Review Board (IRB# IORG0000452-00000767).

### CCLS clinical examination

Details about the measurement of baseline variables in this study cohort and clinical examination have been previously reported.^30^, ^31^, ^32^ A comprehensive clinical exam was conducted by a physician, and complete medical history was taken, in which the participants self-reported personal and family medical histories. Standard laboratory tests were performed on fasting venous blood, including serum glucose, total serum cholesterol, and serum triglycerides, via standardized and automated techniques. Additionally, resting blood pressures were collected using a mercury sphygmomanometer. BMI was calculated for each participant using height and weight values.

### Cardiorespiratory fitness assessment: modified Balke protocol

CRF was measured with a symptom-limited maximal treadmill exercise test using a modified-Balke protocol, which has been described previously.^30^^,^^31^^,^^33^ The CCLS used the modified-Balke protocol, which takes longer than the commonly used Bruce protocol, for CRF testing because it increases workload very gradually, is safer, allows time for more electrocardiography and blood pressure readings, and results in a clearer distribution of fitness categories than the Bruce protocol. With the modified Balke protocol, treadmill speed is initially set to 88 m/min. The grade is set at 0% in the first minute, increases to 2% in the second minute, and continues to increase by 1% every minute subsequently until 25 minutes. At this time, there are no further increases in grade, but the speed of the treadmill increases by 5.4 m/min thereafter until the conclusion of the test. As part of the exercise testing protocol, participants were encouraged to keep their arms free of the hand rests and give their full effort. The point of termination of the test was determined either by the participant from self-reported exhaustion or by the physician for medical concerns. As previously shown, treadmill test time and directly measured maximal oxygen uptake are strongly correlated using this protocol in both women and men (r for women = 0.94, r for men = 0.92).^34^^,^^35^

Individual participant’s treadmill times were categorized into age- and sex-specific categories based on normative treadmill performance data according to standard approaches.^30^^,^^31^ The treadmill time thresholds for CRF quintiles across the age and sex strata developed in the CCLS have been published previously and reported in the Supplemental Table 1.^36^^,^^37^ The categories were then consolidated into mutually exclusive groups: “low fitness,” comprised of participants in the first category, “moderate fitness,” comprised of participants in the second and third categories, and “high fitness,” comprised of participants in the fourth and fifth categories. The use of the Balke protocol allows for the estimation of the participants’ fitness levels in units of metabolic equivalents (METs) using proven and established regression equations.^35^^,^^37^

### Outcome ascertainment: medicare claims data

Medicare claims data were obtained from the Center for Medicare and Medicaid Services (CMS) for CCLS participants eligible for Medicare benefits. The data were obtained from 1999 (the first year that CMS claims data were available) to 2009. The CMS data contain 100% of the claims paid by Medicare for covered health services, allowing tracking of individual participants over time and permitting a consolidated longitudinal view of all utilized health care services by the same individual. The claims data include individual records for each medical service covered by and paid for by Medicare, including date of service, up to 8 separate diagnosis and procedure codes (International Classification of Diseases-9th Revision [ICD-9] code), demographic information, and other data. As reported previously,^38^ incident AF was defined using the Chronic Condition Warehouse algorithm, as determined by ICD-9 Code 427.31 when listed as 2 outpatient claims within 1 year or as a single inpatient claim. This ICD-9 code captures all diagnoses of AF and does not distinguish among paroxysmal, persistent, and permanent AF. This approach to identifying AF has been independently validated and shown to have 94% and 99% sensitivity and specificity, respectively.^39^, ^40^, ^41^, ^42^

### Statistical analysis

Characteristics of the study participants at their midlife exam were summarized by low-, moderate-, and high-fitness categories in the overall cohort and among men and women separately. Crude incidence of AF in Medicare was presented across sex-specific sample deciles of CRF and tested for trend using Wald statistics. The adjusted association between CRF in midlife and risk of AF in older age was assessed using a Cox proportional hazard intensity model. Owing to the unique structure of Medicare claims data, attained age is used as the time scale. Separate models were constructed for categorical (high, moderate, and low [referent] CRF) and continuous measures of CRF and were adjusted for the following covariates—model 1: age, sex, and exam year; model 2: model 1 + BMI; model 3: model 2 + smoking, total cholesterol, baseline systolic blood pressure, smoking status, and glucose. BMI-stratified analyses were also performed to evaluate the association between continuous measures of CRF and risk of AF in obese (BMI ≥30 kg/m^2^) and nonobese individuals separately. In the subset of study participants with CRF assessment on follow-up, the association between change in CRF (CRF visit 2 − CRF visit 1)^21^ and risk of AF was assessed using Cox proportional hazard models that included the covariates described above (model 3) with additional adjustment for change in BMI and time between follow-up and initial visits. Proportional hazards assumptions were confirmed using weighted Schoenfeld residuals.^43^ All statistical analyses were performed using SAS/STAT, version 9.4 (SAS Institute Inc).

## Results

**Figure 1:**
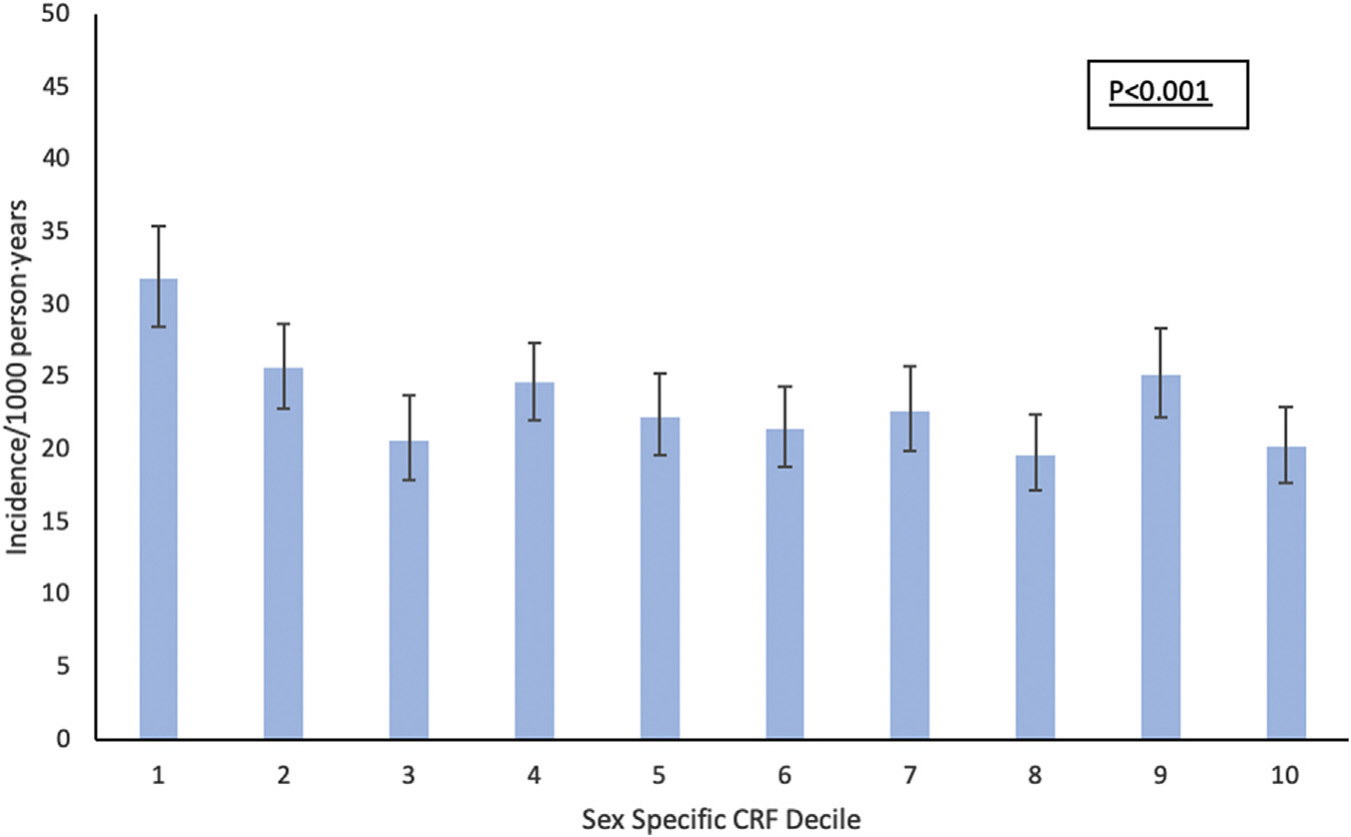
Incidence of Atrial Fibrillation After the Age of 65 Years Across Sex-Specific Deciles of Midlife CRF Among the Participants of Cooper Center Longitudinal Study CRF = cardiorespiratory fitness.

Our study cohort included 18,493 participants (79% men, 98% self-reported White race, median [25th-75th percentile] age: 49.8 years [43.5-56.7], CRF levels: 10.4 METs [9.0-12.2], BMI: 25.3 kg/m^2^ [23.15-27.67], systolic blood pressure: 120 mm Hg [110-130], total cholesterol: 210 mg/dL [187-236]). Compared with the high-CRF group, those in the lower CRF groups were more commonly obese and had a higher burden of traditional cardiovascular (CV) risk factors, including higher blood pressure, higher prevalence of smoking, and diabetes in the overall cohort (Table 1). A similar pattern of clinical characteristics was also observed across the midlife CRF strata in a sex-stratified comparison among men and women separately (Supplemental Tables 2 and 3). During 109,601 person-years of Medicare follow-up, 2,558 incident AF events occurred (women: 364 events [9.2%], men: 2,194 events [15.1%]). The incidence of AF declined from 32 (95% confidence interval [CI]: 28.5-35.4) per 1,000-person years in the lowest sex-specific CRF decile to 20.2 events (95% CI: 17.7-22.9) in the highest decile (Figure 1). In adjusted analyses, higher midlife CRF was associated with a lower risk of AF after adjustment for age, sex, and exam year (Table 2, model 1). However, this association was attenuated and no longer significant after further adjustment for BMI and other risk factors (Table 2, models 2 & 3). A similar pattern of association was observed using continuous measures of CRF in the adjusted models (Table 2). In contrast, higher BMI was significantly associated with a higher risk of AF independent of baseline CRF and other baseline risk factors (hazard ratio [HR] per 1 kg/m^2^: 1.05; 95% CI: 1.03-1.06). In stratified analysis by BMI, the association between continuous measures of CRF and risk of AF was not significant among individuals without obesity (HR per 1 MET: 1.00; 95% CI: 0.98-1.02) as well as those with obesity (HR per 1 MET: 0.99; 95% CI: 0.92-1.06) (*P* interaction CRF∗BMI = 0.06).

**Table 1:** Midlife Characteristics of Participants in the Cooper Center Longitudinal Study by Fitness Categories

	Low Fit (Category 1) (n = 3,008)	Moderate Fit (Category 2-3) (n = 7,447)	High Fit (Category 4-5) (n = 8,038)
Age (y)	47.1 ± 8.6	49.7 ± 8.7	51.7 ± 8.4
Medicare age (y)	70.8 ± 5.2	71.0 ± 5.6	70.7 ± 5.5
Men	2,487 (82.7)	5,985 (80.4)	6,054 (75.3)
BMI (kg/m^2^)	28.0 ± 4.8	26.1 ± 3.6	24.4 ± 2.9
BMI category (kg/m^2^)			
Normal <25	816 ± 27.1	2,867 ± 38.5	4,910 ± 61.1
Overweight 25-29	1,320 ± 43.9	3,609 ± 48.5	2,856 ± 35.5
Obese ≥30	872 ± 29.0	971 ± 13.0	272 ± 3.4
SBP (mm Hg)	123.4 ± 14.9	121.3 ± 14.5	120.7 ± 14.8
Smoker	882 (29.3)	1,351 (18.1)	659 (8.2)
Diabetes	216 (7.2)	276 (3.7)	146 (1.8)
Glucose (mg/dL)	103.4 ± 22.5	100.5 ± 16.4	98.5 ± 12.4
Cholesterol (mg/dL)	219.4 ± 41.2	215.0 ± 39.1	209.1 ± 37.2
CRF (METs)	8.0 ± 1.4	9.8 ± 1.5	12.2 ± 2.2

Values are mean ± SD or n (%).

BMI = body mass index; CRF = cardiorespiratory fitness; MET = metabolic equivalent of task; SBP = systolic blood pressure.

**Table 2:** Multivariable-Adjusted Association Between Midlife CRF Levels, Body Mass Index, and Risk of Atrial Fibrillation in Older Age

	Model 1		Model 2		Model 3	
	HR (95% CI)	*P* Value	HR (95% CI)	*P* Value	HR (95% CI)	*P* Value
CRF categories						
Low fit	Referent group					
Moderate fit	0.88 (0.79-0.98)	0.02	0.97 (0.87-1.09)	0.60	1.00 (0.89-1.12)	0.93
High fit	0.84 (0.75-0.94)	0.002	1.01 (0.89-1.15)	0.84	1.04 (0.92-1.19)	0.52
BMI per kg/m^2^			1.05 (1.04-1.06)	<0.001	1.05 (1.04-1.06)	<0.001
Continuous measure of CRF						
CRF per MET	0.98 (0.96-0.99)	0.01	1.01 (0.99-1.03)	0.29	1.02 (0.99-1.04)	0.15
BMI per kg/m^2^			1.05 (1.04-1.06)	<0.001	1.05 (1.03-1.06)	<0.001

Separate models were constructed using categorical and continuous measures of CRF.

Model 1: adjusted for age, sex, and exam year.

Model 2: Model 1 + BMI.

Model 3: Model 2 + resting systolic blood pressure, cholesterol, glucose, and smoking status.

Low fit = category 1, moderate fit = categories 2 to 3, high fit = categories 4 to 5.

BMI = body mass index; CI = confidence interval; CRF = cardiorespiratory fitness; HR = hazard ratio; MET = metabolic equivalent of task.

An analysis was done on a subset of participants (N = 7,435) with repeat measurement of CRF levels at an average of 2.3 years (SD: 2.8 years) from their midlife examination (Supplemental Table 4). Among these participants, changes in CRF were not associated with the risk of AF in older age (HR [95% CI] per 1 MET increase in CRF: 0.96 [0.90-1.01]; *P* = 0.12; Table 3) after adjustment for midlife measures of BMI, CRF, CV risk factors, and change in BMI over the same period. Change in BMI was also not significantly associated with risk of AF in the most adjusted model (HR [95% CI] per 1 kg/m^2^ increase in BMI: 1.03 [0.97-1.08]; *P* = 0.33) (Table 3).

**Table 3:** Multivariable-Adjusted Association in 7,435 Participants With Multiple Visits Between Baseline and Change Values of Midlife CRF Levels and Body Mass Index on Risk of Atrial Fibrillation in Older Age

	Model 1		Model 2		Model 3	
	HR (95% CI)	*P* Value	HR (95% CI)	*P* Value	HR (95% CI)	*P* Value
Main effects analysis in the subset with change						
Initial CRF per MET	0.98 (0.95-1.02)	0.31	1.03 (0.99-1.06)	0.16	1.02 (0.99-1.06)	0.20
Initial BMI per kg/m^2^	NA		1.06 (1.04-1.09)	<0.001	1.06 (1.03-1.08)	<0.001
Change analysis in the subset with change						
Change in CRF per MET	0.94 (0.89-0.99)	0.03	0.96 (0.90-1.02)	0.14	0.96 (0.90-1.01)	0.12
Change in BMI per kg/m^2^	NA		1.02 (0.97-1.08)	0.38	1.03 (0.97-1.08)	0.33

Model 1: adjusted for age, sex, exam year, baseline CRF, change CRF, and difference in exam years.

Model 2: Model 2 + baseline and change in BMI.

Model 3: Model 3 + resting systolic blood pressure, cholesterol, glucose, and smoking status.

BMI = body mass index; CI = confidence interval; CRF = cardiorespiratory fitness; HR = hazard ratio; MET = metabolic equivalent of task; NA = not available.

## Discussion

The present study demonstrated several important findings related to later-life AF and midlife CRF and BMI. First, lower CRF in midlife was associated with a higher risk of AF in later life. However, this association was primarily driven by differences in BMI and attenuated after adjustment for the same. Second, higher BMI in midlife was significantly associated with a higher risk of AF after the age of 65 years, independent of other midlife risk factors and CRF. Third, intermediate-term changes in CRF over an average of 2.3 years of follow-up were not significantly associated with the risk of later-life AF. Our study findings highlight the independent contributions of midlife CRF and BMI to the risk of AF in older age (Central Illustration).

**Central Illustration:**
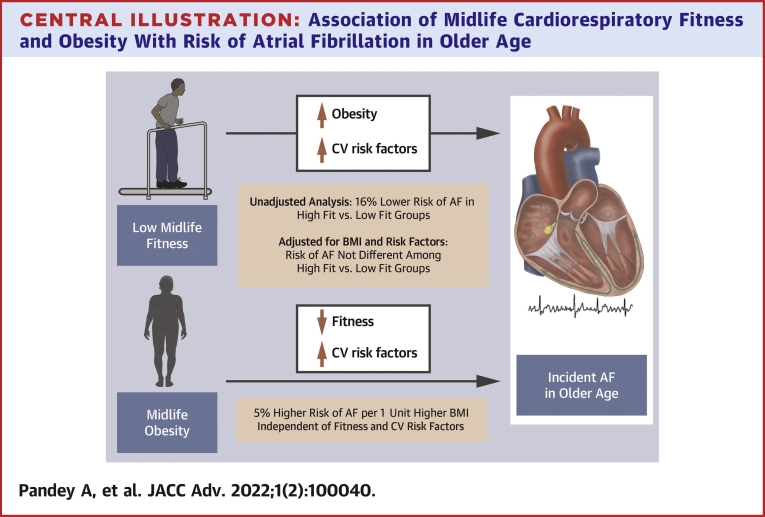
Association of Midlife Cardiorespiratory Fitness and Obesity With Risk of Atrial Fibrillation in Older Age The association of low midlife fitness with higher risk of incident atrial fibrillation (AF) is driven by higher burden of obesity and cardiovascular (CV) risk factors in less fit individuals. In contrast, the association between obesity in midlife and risk of AF is independent of fitness levels and CV risk factor burden. BMI = body mass index.

Previous studies have evaluated the association between CRF and the risk of AF with inconsistent findings. Some studies from European cohorts have demonstrated a U-shaped association between CRF and the risk of AF.^22^^,^^27^^,^^28^ In a Finnish cohort study, Khan et al^27^ observed the lowest risk of AF among individuals with CRF levels between 6 and 9 METs and an increased risk at the lower and higher ends of CRF distribution. Similarly, in a cohort of Swedish men, a U-shaped association was noted between CRF in young adulthood and risk of AF in middle age.^22^ In contrast, studies from the United States in cohorts of older individuals who underwent clinically indicated stress tests have demonstrated a linear, inverse association between CRF levels and risk of AF.^23^^,^^24^^,^^26^^,^^44^ In the present study, we also observed that a higher midlife CRF was associated with a lower risk of AF. However, this association was largely driven by differences in BMI and was attenuated after adjustment for the same.

The discordant findings between our study and others may be related to study population and design differences. For example, prior studies from US cohorts were limited by the referral nature of the study population,^24^^,^^26^^,^^44^ the use of nonexercise test-based estimated measures of CRF,^23^ or inadequate adjustment for confounders such as BMI.^23^^,^^44^ Furthermore, most U.S. cohort studies evaluated the association between CRF assessed in older age and downstream risk of AF over the next few years. Thus, the observed associations between higher CRF and lower risk of AF in these studies are also susceptible to reverse causation. In contrast, our study was conducted among community-based, relatively healthy individuals who underwent symptom-limited maximal treadmill test in midlife and had AF outcomes assessed decades later, minimizing the potential for referral bias and reverse causation bias. Our study findings add to the existing literature by evaluating the independent associations of objectively measured CRF and BMI in midlife and the risk of AF in older age.

Our study also evaluated the association between short-term longitudinal changes in CRF and the risk of AF in later life. We observed that these changes in CRF and BMI were not associated with the risk of AF. Consistent with our observation, Khan et al^27^ also did not observe a significant association between changes in CRF and risk of AF in a cohort of Finnish participants. In contrast to our observations, Garnvik et al^25^ demonstrated that a 1-MET increase in estimated CRF levels was associated with a 10% lower risk of AF. However, Garnvik et al^25^ used non–exercise-based estimates of CRF and did not adjust for baseline BMI in the models evaluating the association of CRF change with the risk of AF.

The association between CRF and the risk of AF noted in this study is different from that observed for CV outcomes. Previous studies from the CCLS and other cohorts have demonstrated that a higher CRF is associated with a significantly lower risk of CVD, including myocardial infarction, stroke, and HF.^19^^,^^21^^,^^38^^,^^45^^,^^46^ Furthermore, improvements in CRF have been associated with a lower risk of CVD, such as HF.^20^^,^^21^ In the present study, we observed that the higher CRF related to lower risk of AF was largely driven by BMI. The differences in how CRF may influence the risk of AF vs other CV conditions may be related to the effect of exercise and higher CRF on the cardiac structure. Specifically, left atrial (LA) dilation is considered 1 of the key cardiac substrates that underlie the development of AF. While greater exercise and high CRF are associated with a lower burden of traditional CV risk factors, it is also associated with LA dilation.^47^, ^48^, ^49^, ^50^, ^51^, ^52^, ^53^ Thus, the favorable downstream effects of lowering the CV risk factor burden in preventing AF may be counterbalanced by dilating LA in high-fit individuals.

In contrast with CRF, we observed a significant association between BMI in midlife and risk of AF in older age, independent of CRF and other risk factors. Several prior studies have demonstrated that obesity is an independent risk factor for AF.^7^^,^^9^^,^^14^^,^^54^ However, unlike the present study, most previous studies did not account for the contribution of CRF to the association between BMI and AF. The findings from the present study highlight the primacy of obesity over low CRF for the development of AF in older individuals.

Obesity may promote the development of AF via systemic and local pathways. Excess adipose tissue alters the metabolic, neurohormonal, and inflammatory milieu of the CV system, resulting in volume expansion, myocardial fibrosis, and autonomic dysfunction.^55^ Furthermore, epicardial and pericardial fat accumulation may induce local changes to the myocardium leading to structural abnormalities such as LA enlargement.^55^ Consistently, LA size has been shown to attenuate the obesity-associated risk of AF in epidemiological studies.^9^

Our observations may have implications for the primary prevention of AF. The present study findings suggest that a higher BMI in middle age may be a more important and independent risk factor for AF than CRF. Prior studies with intensive lifestyle interventions targeting CRF improvement and modest weight loss have failed to demonstrate a significant reduction in the risk of AF.^56^ In contrast, aggressive weight-loss strategies, such as bariatric surgery, have been shown to lower the AF risk significantly.^57^ Such aggressive weight-loss strategies need to be tested in prospective, randomized controlled trials to determine the optimal approaches for the prevention of AF.

### Study limitations

Our study is not without limitations. First, the participants of the CCLS were predominantly men of the White race and had high income and education levels. The skewness in the sex distribution limits our ability to evaluate risk factors for AF among women. While the exact information on income is not available in the CCLS, participants are generally described as coming from middle to upper socioeconomic strata with access to health care. Participants are also well-educated, with an average of 16 years of education (SD 3) among the ∼20% reporting. Furthermore, the study participants have higher levels of CRF, lower BMI, and lower burden of traditional CV risk factors than the general US population. Thus, the study findings may have limited generalizability among women, individuals of minority race/ethnicity, socioeconomically disadvantaged individuals, and those with a high burden of CV risk factors. Second, due to the limited availability of these data, income and education level are not reported in Table 1. Third, incident AF was captured using Medicare administrative claims data, making our outcome assessment susceptible to misclassification errors. Furthermore, the ICD-9 code for AF cannot distinguish among paroxysmal, persistent, and permanent AF, limiting our ability to identify specific AF types. However, the sensitivity and specificity of Medicare claim to identify AF are very high, limiting such misclassification's magnitude.^38^, ^39^, ^40^, ^41^ Fourth, CRF was not assessed using the gold standard of directly measured peak oxygen uptake in the CCLS. However, prior reports have demonstrated a strong correlation between directly measured peak oxygen uptake and treadmill speed- and grade-based CRF levels (r for men = 0.92, r for women = 0.94). Fifth, echocardiographic data are not available at the baseline visit for most study participants, limiting our ability to evaluate how LA size and function may modify the association of BMI and CRF with the risk of AF. Finally, given the observational design of our study, results may be susceptible to residual confounding.

## Conclusions

Among participants in the CCLS, baseline CRF and improvement in CRF in midlife were not associated with incident AF after accounting for differences in BMI. In contrast, a higher BMI in midlife was associated with a higher risk of AF, independent of AF risk factors and CRF. These findings suggest that obesity, but not low CRF, in midlife may be an important driver of the higher burden of AF in older individuals.

PERSPECTIVES **COMPETENCY IN MEDICAL KNOWLEDGE:** Obesity but not low fitness in midlife is independently associated with the risk of AF in older age.

**TRANSLATIONAL OUTLOOK:** Future studies are needed to determine if lifestyle interventions aimed at intentional weight loss may lower the risk of AF in older age.

## Funding support and author disclosures

Dr Pandey has received grant funding outside the present study from Applied Therapeutics and 10.13039/100005564Gilead Sciences; has received honoraria outside of the present study as an advisor/consultant for Tricog Health Inc, Lilly USA, Rivus, and Roche Diagnostics; has received nonfinancial support from 10.13039/100004319Pfizer and 10.13039/100004334Merck; and has received research support for this study from the Texas Health Resources Clinical Scholarship, the 10.13039/100005564Gilead Sciences Research Scholar Program, and the 10.13039/100000049National Institute on Aging GEMSSTAR Grant (1R03AG067960-01). All other authors have reported that they have no relationships relevant to the contents of this paper to disclose.
