# Supplementary material for: Mid-Life Cardiorespiratory Fitness, Obesity, and Risk of Atrial Fibrillation: The Cooper Center Longitudinal Study
Source: JACC Adv. 2022 May 31;1(2):100040. doi: 10.1016/j.jacadv.2022.100040 (PMC11198193; doi:10.1016/j.jacadv.2022.100040)

**Supplemental Table 1:** Balke Protocol Treadmill Time Thresholds* for Fitness Quintiles Across Age Strata Developed in the Cooper Center Longitudinal Study as reported previously^1^

| **Age Group** | **Quintile 1** | **Quintile 2** | **Quintile 3** | **Quintile 4** | **Quintile 5** |
| --- | --- | --- | --- | --- | --- |
| **Men** | | | | | |
| 20-39 years | ≤15.00 | 15.02, 18.00 | 18.02, 20.33 | 20.35 23.58 | ≥23.60 |
| 40-49 years | ≤13.50 | 13.52, 16.05 | 16.07, 19.00 | 19.02, 22.02 | ≥22.03 |
| 50-59 years | ≤11.00 | 11.02, 13.58 | 13.60, 16.00 | 16.02, 19.17 | ≥19.18 |
| ≥60 years | ≤7.75 | 7.77, 10.50 | 10.52, 13.07 | 13.08, 16.35 | ≥16.37 |
| **Women** | | | | | |
| 20-39 years | ≤10.27 | 10.28, 13.00 | 13.02, 15.00 | 15.02, 18.00 | ≥18.02 |
| 40-49 years | ≤8.93 | 8.95, 11.00 | 11.02, 13.03 | 13.05, 16.02 | ≥16.03 |
| 50-59 years | ≤6.95 | 6.97, 9.00 | 9.02, 10.68 | 10.70, 13.23 | ≥13.25 |
| ≥60 years | ≤5.47 | 5.48, 7.00 | 7.02, 9.00 | 9.02, 11.25 | ≥11.27 |
| * Time on treadmill in minutes at volitional exhaustion.  ^1.^ Sui X, LaMonte MJ, Laditka JN, Hardin JW, Chase N, Hooker SP, Blair SN. Cardiorespiratory fitness and adiposity as mortality predictors in older adults. *JAMA.* 2007;298(21):2507-2516. | | | | | |

**Supplemental Table 2:** Midlife characteristics of women participants stratified by their cardiorespiratory fitness levels

| **Participant Characteristics** | **Low Fit**  **(Category 1)**  **(n=521)** | **Moderate Fit**  **(Category 2-3) (n=1,462)** | **High Fit**  **(Category 4-5) (n=1,984)** |
| --- | --- | --- | --- |
| Age (years) | 49.3 (9.4) | 51.4 (9.1) | 53.4 (8.2) |
| Medicare Age (years) | 71.4 (5.8) | 71.0 (5.8) | 71.8 (6.5) |
| BMI, (kg/m^2^) | 25.2 (5.3) | 23.7 (3.9) | 22.4 (2.9) |
| SBP, (mm Hg) | 119 (16) | 117 (16) | 117 (16) |
| Glucose (mg/dL) | 97 (16) | 96 (13) | 95(12) |
| Cholesterol (mg/dL) | 214 (39) | 214 (40) | 210 (37) |
| CRF (METs) | 6.3 (0.86) | 7.9 (0.97) | 10.0 (1.5) |
| Data presented as Mean (Standard deviation) for continuous variables and n (%) for categorical variables. CRF: Cardiorespiratory fitness; MET: Metabolic equivalent of Task; BMI: Body mass index; SBP: Systolic blood pressure | | | |

**Supplemental Table 3:** Midlife characteristics of men participants stratified by their cardiorespiratory fitness levels

| **Participant Characteristics** | **Low Fit**  **(Category 1)**  **(n=2,487)** | **Moderate Fit**  **(Category 2-3) (n=5,985)** | **High Fit**  **(Category 4-5) (n=6,054)** |
| --- | --- | --- | --- |
| Age (years) | 46.6 (8.3) | 49.2 (8.6) | 51.2 (8.4) |
| Medicare Age (years) | 70.7 (5.1) | 71.0 (5.6) | 70.9 (5.6) |
| BMI, (kg/m^2^) | 28.6 (4.5) | 26.7 (3.2) | 25.0 (2.5) |
| SBP, (mm Hg) | 124 (15) | 122 (14) | 122 (14) |
| Glucose (mg/dL) | 105 (23) | 102 (17) | 100 (12) |
| Cholesterol (mg/dL) | 221 (42) | 215 (39) | 209 (37) |
| CRF (METs) | 8.4 (1.2) | 10.3 (1.2) | 12.9 (1.8) |
| Data presented as Mean (Standard deviation) for continuous variables and n (%) for categorical variables. CRF: Cardiorespiratory fitness; MET: Metabolic equivalent of Task; BMI: Body mass index; SBP: Systolic blood pressure | | | |

**Supplemental Table 4**: Midlife characteristics of study participants with repeat cardiorespiratory fitness test on follow up

| **Participant characteristics** | **Men  (n=6301)** | **Women**  **(n=1134)** |
| --- | --- | --- |
| Age at initial visit (years) | 48.6 (8.1) | 50.8 (8.5) |
| Age at follow-up visit (years) | 50.9 (8.2) | 53.6 (8.5) |
| Change in age (years) | 2.3 (2.9) | 2.8 (3.1) |
| Initial CRF (METs) | 11.4 (2.2) | 9.1 (1.8) |
| Change in CRF (METs) | 0.4 (1.3) | 0.3 (1.2) |
| Initial SBP (mm Hg) | 121.9 (13.6) | 115.9 (14.8) |
| Change in SBP (mm Hg) | -1.2 (12.8) | 0.5 (13.5) |
| Initial BMI (kg/m^2^) | 25.9 (3.1) | 22.7 (3.3) |
| Change in BMI (kg/m^2^) | -0.06 (1.4) | 0.1 (1.7) |
| Data presented as Mean (Standard deviation) for continuous variables and N (%) for categorical variables. CRF: Cardiorespiratory fitness; MET: Metabolic equivalent of Task; BMI: Body mass index; SBP: Systolic blood pressure | | |

**Supplemental Figure 1:** Consort Diagram for the Study


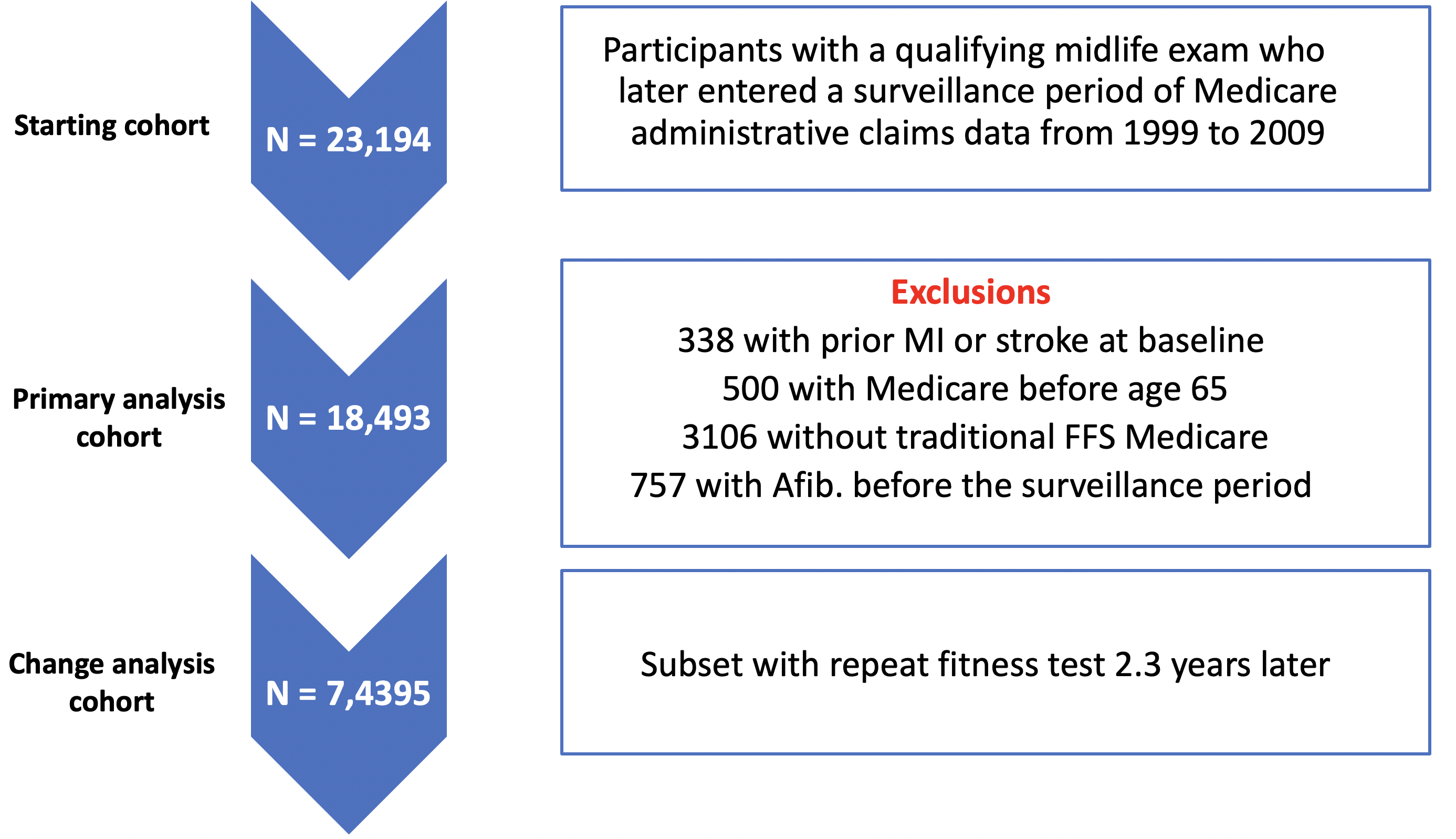

Supplement: Supplemental Figure 1 and Tables 1-4 [file mmc1.docx]
